# Design of the Dutch multicentre study on opportunistic screening of geriatric patients for atrial fibrillation using a smartphone PPG app: the Dutch-GERAF study

**DOI:** 10.1007/s12471-024-01868-6

**Published:** 2024-04-15

**Authors:** Dutch-GERAF investigators, Lennaert A. R. Zwart, Jocelyn R. Spruit, Martin E. W. Hemels, Joris R. de Groot, Ron Pisters, Robert K. Riezebos, René W. M. M. Jansen

**Affiliations:** 1 Department of Geriatric Medicine, Dijklander Hospital, Hoorn, The Netherlands; 2 Department of Geriatric Medicine, Northwest Hospital, Alkmaar, The Netherlands; 3 Aging and Later Life, Amsterdam Public Health, Amsterdam University Hospital, Amsterdam, The Netherlands; 4 Department of Cardiology, Rijnstate Hospital, Arnhem, The Netherlands; 5 Department of Cardiology, Radboud University Hospital, Nijmegen, The Netherlands; 6 Department of Cardiology, Amsterdam University Hospital, Amsterdam, The Netherlands; 7 Heart Centre, Department of Cardiology, OLVG, Amsterdam, The Netherlands

**Keywords:** Opportunistic screening, Frailty, eHealth, Photoplethysmography, Geriatrics, Atrial fibrillation

## Abstract

**Background:**

Screening of high-risk patients is advocated to achieve early detection and treatment of clinical atrial fibrillation (AF). The Dutch-GERAF study will address two major issues. Firstly, the effectiveness and feasibility of an opportunistic screening strategy for clinical AF will be assessed in frail older patients and, secondly, observational data will be gathered regarding the efficacy and safety of oral anticoagulation (OAC).

**Methods:**

This is a multicentre study on opportunistic screening of geriatric patients for clinical AF using a smartphone photoplethysmography (PPG) application. Inclusion criteria are age ≥ 65 years and the ability to perform at least three PPG recordings within 6 months. Exclusion criteria are the presence of a cardiac implantable device, advanced dementia or a severe tremor. The PPG application records patients’ pulse at their fingertip and determines the likelihood of clinical AF. If clinical AF is suspected after a positive PPG recording, a confirmatory electrocardiogram is performed. Patients undergo a comprehensive geriatric assessment and a frailty index is calculated. Risk scores for major bleeding (MB) are applied. Standard laboratory testing and additional laboratory analyses are performed to determine the ABC-bleeding risk score. Follow-up data will be collected at 6 months, 12 months and 3 years on the incidence of AF, MB, hospitalisation, stroke, progression of cognitive disorders and mortality.

**Discussion:**

The Dutch-GERAF study will focus on frail older patients, who are underrepresented in randomised clinical trials. It will provide insight into the effectiveness of screening for clinical AF and the efficacy and safety of OAC in this high-risk population.

**Trial registration:**

NCT05337202.

## Background

Guidelines recommend screening for atrial fibrillation (AF) [1, 2]. Patients visiting a geriatric outpatient clinic are frail and known to have a high prevalence and a high incidence of AF [3, 4]. A recent cost-effectiveness evaluation showed the benefit of opportunistic screening for AF in the geriatric outpatient population [5]. However, robust data on treatment of frail patients with AF are lacking, since frailty is poorly measured and reported in large randomised clinical trials (RCTs), and it remains unclear to what extent frail patients were included [6–10]. Therefore, more knowledge about effective screening for AF as well as the safety and efficacy of anticoagulation is required, especially in frail older patients.

Different screening strategies have been studied, from single time-point screening to continuous monitoring [4, 11–17]. The LOOP study and the NOAH-AFNET 6 trial showed that continuous screening is very effective but could lead to false-positive case findings, and treatment of subclinical AF might be harmful [11, 12]. The clinical relevance of screen-detected cases is the subject of an ongoing discussion. Here the difference between clinical and subclinical AF seems to be the culprit, since they have a different stroke risk [16].

## Methods

### Setting and design

**Fig. 1:**
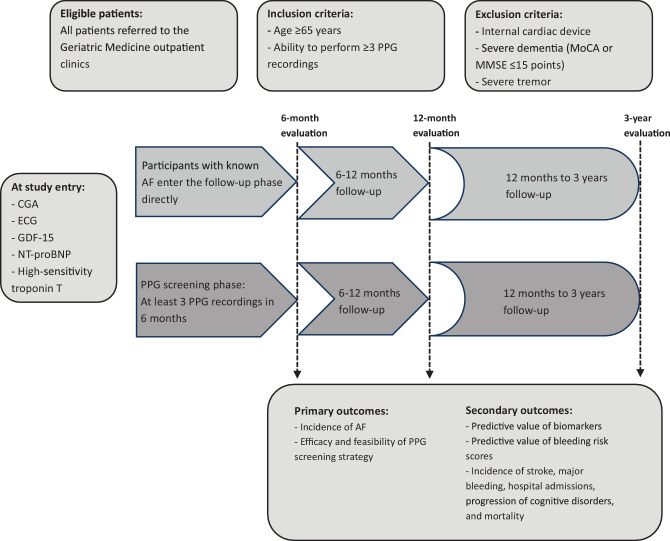
Dutch-GERAF study protocol. (*PPG* photoplethysmography, *MoCA* Montreal Cognitive Assessment, *MMSE* Mini Mental State Examination, *CGA* Comprehensive Geriatric Assessment, *ECG* electrocardiography, *GDF-15* growth/differentiation factor 15, *NT-proBNP* N-terminal pro-brain natriuretic peptide, *AF* atrial fibrillation)

The Dutch-GERAF study is a prospective, multicentre, observational cohort study, divided into a 6-month phase of opportunistic screening of geriatric patients for AF using a smartphone photoplethysmography (PPG) application, and a 30-month observational follow-up phase. By means of (repeated) single-time-point screening followed by electrocardiographic confirmation of AF, the chance of diagnosing subclinical AF is minimised. The total duration of the study will be 3 years per patient. The study will be performed in the geriatric outpatient units of six large, non-academic teaching hospitals. A graphical representation of the study design is shown in Fig. 1.

### Consent, ethical approval, privacy, and protection of data

The study protocol was evaluated and approved by the Medical Ethics Committee Oost Nederland at Radboud University in Nijmegen, The Netherlands, with reference number 2019-5889. Patients receive written information about the study before their physician’s appointment and have the opportunity to ask questions during their appointment before deciding to participate or not. All patients included in the study will provide written informed consent or have a legal representative sign on their behalf. Data will be collected through the clinical data management platform Castor.

### Inclusion and exclusion

Inclusion criteria are age ≥ 65 years and the ability to perform ≥ 3 PPG recordings within 6 months. Exclusion criteria are an implanted cardiac device, a severe tremor (as performing reliable PPG recordings is expected to be too challenging), or severe dementia, defined as a Montreal Cognitive Assessment or Mini Mental State Examination score of 15 points or less. We aim to enrol 1000 patients into the cohort, based on a feasibility estimation for all participating centres during an inclusion period of 2 years or until 1000 participants are enrolled.

### Primary and secondary outcomes

The primary outcome of the 6‑month screening phase is the incidence of clinical AF, defined as newly detected AF confirmed on a 12-lead electrocardiogram (ECG). It is possible that the PPG application will register a short episode of paroxysm AF, which at the time of the 12-lead ECG can no longer be confirmed. These cases will not be classified as newly detected clinical AF. The chance of detecting subclinical AF is almost negligible by means of ECG confirmation. In addition to screening-detected and ECG-confirmed AF, newly detected AF on the 12 lead ECG at study entry, or on any other ECG performed during usual care, will be counted as newly detected clinical AF. It is expected that anticoagulation will be initiated for all patients diagnosed with AF or atrial flutter and, following the ESC guideline, a new oral anticoagulant will be prescribed [1].

The primary outcome of the observational 1‑ and 3‑year follow-up phase is the rate of major bleeding following the ISTH criteria [18]. External validation will take place of the HAS-BLED, ATRIA, ORBIT and the ABC-bleeding risk score [19–22]. Risks and rates will be calculated overall, and separately for patients with sinus rhythm who remain in sinus rhythm over the course of the study, for patients with clinical AF at baseline, and for patients with newly detected clinical AF.

The secondary outcome is the feasibility of this PPG screening strategy for frail outpatients. The predictive value of N‑terminal pro-brain natriuretic peptide (NT-proBNP) for newly detected clinical AF will be calculated as an area under the ROC curve, and as an odds ratio using a cut-off of 125 pg/l, comparable to the STROKESTOP 2 study [13]. The association will be determined between NT-proBNP, growth/differentiation factor 15 (GDF-15) and high-sensitivity troponin T and frailty. Furthermore, we aim to identify baseline patient characteristics associated with unplanned hospital admissions, transient ischaemic attacks, ischaemic and thromboembolic stroke, and mortality at 1 and 3 years’ follow up.

### Baseline and demographic data

Patients will undergo a comprehensive geriatric assessment (CGA), which is a multidisciplinary approach that examines factors that contribute to frailty, resilience, cognition, functional state, evaluation of polypharmacy, and evaluation of mobility. The assessment evaluates all available medical data on co-morbidities, previous investigations, and includes an outpatient clinic blood pressure measurement. The CGA incorporates standard laboratory testing and other tests specific to the individual patient. Cognitive function is always evaluated, and when expected to be diminished, either cognitive screening or a full neuropsychological assessment will be performed [23].

Frailty will be assessed at baseline with a CGA-based frailty index (FI), following the accumulation of deficits model [24, 25]. The index will consist of 46 factors, assigned a weight of 1 or 2, and is calculated as: factors absent, 0 points; factors present, 1 or 2 points. The total number of points is calculated, and the index is calculated as the total points per patient/maximum (51 points). A value of 0.18 or higher corresponds to moderate frailty, and a value of 0.24 or higher to severe frailty [4, 26]. The list of factors and weights in the FI are described in Tab. 1.

**Table 1:** Factors and weights in the frailty index

*Somatic*	*Weight*	*Psychological*	*Weight*
Underweight (BMI < 20)	1	Depression	1
Obesity (BMI > 30)	1	Mild cognitive impairment	1
Former smoker	1	Dementia, of any aetiology	2
Atrial fibrillation	1		
Active smoker	2	*Functional*	*Weight*
Alcohol, more than 2 units/day	1	ADL with help	1
Hypertension	1	ADL dependent	2
Heart failure	1	iADL with help	1
Stroke, 1 or multiple	1	iADL dependent	2
Angina pectoris and/or myocardial infarct	1		
Hypercholesterolaemia	1	Visual impairment	1
Diabetes mellitus	1	Hearing impairment	1
Liver cirrhosis	1	Arthrosis	1
Peripheral arterial disease	1	Chronic pain	1
COPD	1	Orthostatic hypotension	1
Asthma	1	Weak or decreased handgrip	1
OSAS	1	Parkinsonism	1
Malignancy	1	Gait disturbance	1
Gout	1	Falls	1
Osteoporosis	1	Walking device	1
Polyneuropathy	1		
Parkinson disease	1	*Social*	*Weight*
Polypharmacy (6 different prescription drugs or more)	1	Alone or widowed	1
Impaired kidney function (eGFR 30–50 ml/min)	1	Living with assistance	1
Severely impaired kidney function (eGFR under 30 ml/min)	2	Nursing home	2
Total	27	Total	24
*Overall total*	*51*		

*BMI* body mass index, *COPD* chronic obstructive pulmonary disease, *OSAS* obstructive sleep apnoea syndrome, *eGFR* estimated glomerular filtration rate, *ADL* activities of daily living, *iADL* instrumental activities of daily living

### Opportunistic screening protocol

At study entry, all patients undergo a 12-lead ECG. The PPG application is installed, and patients are instructed on how to use the application. The screening protocol is designed to cause minimal interference in usual care. Opportunistic screening will last for 6 consecutive months after inclusion. At every appointment, patients perform a PPG recording. Furthermore, patients are stimulated to perform weekly PPG recordings themselves. Patients have the liberty to perform PPG recordings more frequently. The recordings last for 90 s and are performed at the tip of the patient’s index finger. If the quality of the signal is too low, the application asks the user to perform another recording.

This study uses the Heart Rhythm Software Development Kit (SDK) PPG application [27, 28] developed by Happitech (Rotterdam, The Netherlands), which does not collect any other data. The Heart Rhythm SDK algorithm automatically classifies the recording into a regular rhythm, or an irregular rhythm suspected to be AF, based on the tachogram and Poincaré plot [28]. In the validation study, the sensitivity for AF detection of the Heart Rhythm SDK has been reported to be 96.3% (95% confidence interval 90.8–99.0%), the specificity 93.5% (95% confidence interval 87.1–97.4%), positive predictive value 93.3% and negative predictive value 93.5% [28].

### Laboratory protocol

During standard blood sampling, two additional lithium-heparin tubes will be collected, centrifuged and stored at −20 °C. Stored blood samples will be analysed at Dijklander Hospital (Hoorn, The Netherlands). There, the study-specific analysis will be performed: GDF-15, NT-proBNP and high-sensitivity troponin T. If the treating physician sees no reason for laboratory testing, patients will not be exposed to blood sampling for the study only, and missing data is accepted. Laboratory testing that is considered standard care will be performed at the participating centre, with local procedures and equipment.

### Expected loss to follow-up

The expected drop-out rate as regards participation in and execution of the screening protocol is 5–10%. Both the results of screening as an ‘intention-to-screen’ as well as for patients that fully followed the protocol will be reported. Patients are expected to be lost to follow-up mainly because of mortality, with a rate of 3% per year. Patients lost to follow-up for other reasons will be censored at their last visit.

### Expected incidence of outcome measures

The expected baseline prevalence of AF of geriatric patients is 20%. Our previous single-centre screening study found a 5.5% annual incidence of clinical AF by means of (repeated) single-time-point screening [4]. Since the GERAF study has a screening phase of 6 months, an incidence of 2.7% new clinical AF cases is expected [4].

For an estimation of the number of major bleeding episodes over the 3‑year follow-up period, we use the risk of major bleeding per 100 treatment years with oral anticoagulation (OAC). Within the cohort, the estimated number of patients with AF at baseline is 200, and newly diagnosed AF is expected in 25 patients. Recent analyses show that the risk of major bleeding in geriatric patients with clinical AF is lower than that predicted by the HAS-BLED score [29]. Our assumption is that in the GERAF cohort a bleeding rate of between 2 and 6% per year will be observed during the total follow-up period of 3 years. We recently reported an 87% OAC adherence in this patient category, compared to approximately 60% reported earlier by Tulner and colleagues [4, 30].

If 90% of patients with AF use OAC, this will concern 203 patients (90% of 200 patients known to have AF and 25 patients with newly diagnosed AF). With a loss to follow-up rate of 3% and a rate of OAC discontinuation of 5% per year, the expected number of treatment years will be:

$$(1\mathrm{st\ }\text{year of follow-up})+(2\mathrm{nd\ }\text{year of follow-up})+(3\mathrm{rd\ }\text{year of follow up})=203\times 0.92+188\times 0.92+172\times 0.92=518\,\text{years on OAC}$$

The number of expected major bleeding events among anticoagulated patients over the course of the 3‑year follow-up will range from 10 to 31, based on the following estimation:

$$518\,\text{treatment years}\times \text{bleeding rate of}\,2/100\,\text{treatment years}=10.4\,\text{major bleeding events}{,}\mathrm{to}$$

$$518\,\text{treatment years}\times 6/100\,\text{treatment years}=31.1\,\text{major bleeding events}$$

The cohort will consist of a heterogenous population, and therefore an estimation of the incidence of the secondary outcomes will have a very wide range and be not very informative. The observational phase of the GERAF study, however, could be helpful in the future design and power calculation of studies focusing on the frail older population.

### Statistical analysis plan

Continuous outcome variables will be explored and assessed for normality. For non-normally distributed outcome variables, appropriate transformation will be applied. Categorical variables will be compared using the chi-squared test. An assessment of the association of baseline characteristics with newly detected AF will be performed by binary logistic regression analysis, with appropriate adjustment and reporting of the odds ratio (OR) with 95% confidence interval (95% CI).

For the evaluation of the risk of major bleeding, a multilevel analysis with a logistic regression model will be performed, with appropriate adjustment and reporting of the OR and 95% CI. For the performance of the bleeding risk scores a multilevel analysis and Cox regression analysis based on the standard cut-off scores will be performed for each bleeding risk score, with reporting of the OR and 95% CI for major bleeding for each score. Furthermore, an area under the ROC curve will be calculated for each bleeding risk score. Final analysis and validity of the results will be checked and discussed with a statistician.

## Discussion

The GERAF study will include frail older patients with a broad spectrum of co-morbidities and cognitive dysfunction. It will provide insight into the effectiveness of a patient-initiated eHealth strategy for the detection of clinical AF by repeated single-time-point screening and the anticoagulation-related bleeding risk in a frail population.

Strengths are the detailed assessment of functional and cognitive state, and a CGA-based FI. The CGA-based FI provides a more precise assessment and classification of frailty in comparison to bed-side frailty screeners. Furthermore, this determination of frailty will make comparison with patients’ frailty in other cohorts possible [24, 26]. The large sample size and recruitment at multiple centres will contribute to the generalisability of the results.

Since the risk of major bleeding increases with age, the large proportion of participants without anticoagulation in this cohort will make it possible to appreciate the additive risk of anticoagulation, over and above the higher risk of bleeding already expected in this cohort, and to relate this to their frailty.

The main limitation of the study will be that causality cannot be assessed, since the study is observational and does not randomise or assign patients to an intervention or control arm. An inclusion bias is possible.

In conclusion, the GERAF study will assess a PPG-based screening strategy to detect clinical AF in a geriatric population. It will provide a better understanding of the efficacy and safety of OAC in a large cohort of frail elderly patients, including patients with cognitive dysfunction, who are traditionally underrepresented in randomised clinical trials.
